# Supplementary material for: Identification of photoperiod-induced specific miRNAs in the adrenal glands of Sunite sheep (Ovis aries)
Source: Front Vet Sci. 2022 Jul 22;9:888207. doi: 10.3389/fvets.2022.888207 (PMC9354845; doi:10.3389/fvets.2022.888207)
Supplement: Supplementary file 1 [file Data_Sheet_1.ZIP › Supplementary file/miRDeep2 file.zip!/miRDeep2 file/Novel_Structure/SP42A1/Novel_514.pdf]

Provisional ID	:	NC_019483.2_25635
Score total	:	13.1
Score for star read(s)	:	3.9
Score for read counts	:	5.7
Score for mfe	:	1.9
Score for randfold	:	1.6
Score for cons. seed	:	
Total read count	:	23
Mature read count	:	22
Loop read count	:	0
Star read count	:	1

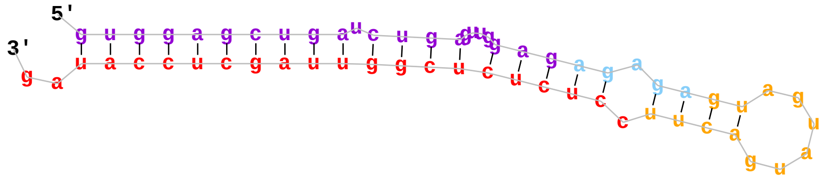

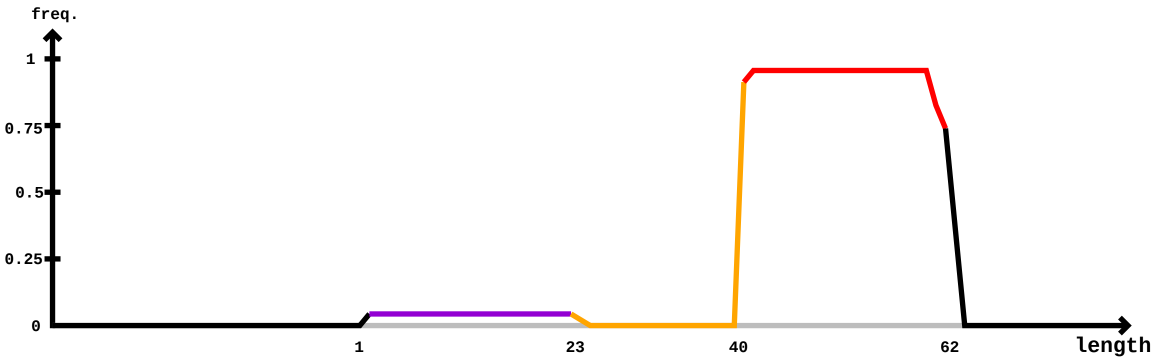


```
                                 Star                                       Mature
5'- uuuacucaguucacugaccgauugugccaguguggagcugaucugaguuggagagagaguaguaugacuuccucucucgguuagcuccauagauggcagaucagccuucu  -3'  obs
    uuuacucaguucacugaccgauugugccaguguggagcugaucugaguuggagagagaguaguaugacuuccucucucgguuagcuccauagauggcagaucagccuucu       exp
    .....((((....))))..((((.(((((.(((((((((((.((((....(((((.((((......)))).))))))))))))))))))))..)))))))))........  reads  mm  sample
    ..............................guggagcugaucugaguuggag.........................................................  1      0   seq
    ....................................................................ccucucucgguuagcuccau.....................  3      0   seq
    ....................................................................ccucucucgguuagcuccaua....................  2      0   seq
    ....................................................................ccucucucgguuagcuccauag...................  15     0   seq
    ....................................................................ccucucucgguuagcuccauaga..................  1      0   seq
    .....................................................................cucucucgguuagcuccauag...................  1      0   seq
```
